# Verification of the Necessity of the Tolyl Group of PF-543 for Sphingosine Kinase 1 Inhibitory Activity

**DOI:** 10.3390/molecules25112484

**Published:** 2020-05-27

**Authors:** Su Bin Kim, Taeho Lee, Hong Seop Moon, Sung Hwan Ki, Yoon Sin Oh, Joo-Youn Lee, Sang-Bum Kim, Jeong-Eun Park, Yongseok Kwon, Sanghee Kim, Dong Jae Baek, Eun-Young Park

**Affiliations:** 1 College of Pharmacy, Mokpo National University, Jeonnam 58554, Korea; rlatnqls0801@naver.com (S.B.K.); hbsmoon@mokpo.ac.kr (H.S.M.); 2 College of Pharmacy, Research Institute of Pharmaceutical Sciences, Kyungpook National University, Daegu 41566, Korea; tlee@knu.ac.kr; 3 College of Pharmacy, Chosun University, Gwangju 61452, Korea; shki@chosun.ac.kr; 4 Department of Food and Nutrition, Eulji University, Seongnam 13135, Korea; ysoh@eulji.ac.kr; 5 Chemical Data-Driven Research Center, Korea Research Institute of Chemical Technology, 141 Gajeong-ro, Yuseong-gu, Daejeon 34114, Korea; leejooyoun@snu.ac.kr; 6 New Drug Development Center, Daegu-Gyeongbuk Medical Innovation Foundation, 80 Cheombok-ro, Dong-gu, Daegu 41061, Korea; ksb2014@dgmif.re.kr (S.-B.K.); parkje6605@dgmif.re.kr (J.-E.P.); 7 Department of Chemistry, Sogang University, Seoul 04107, Korea; ykwon@sogang.ac.kr; 8 College of Pharmacy, Seoul National University, Seoul 08826, Korea; pennkim@snu.ac.kr

**Keywords:** sphingosine kinase, PF-543, BODIPY, anticancer, inhibitor, derivative

## Abstract

PF-543, the most potent sphingosine kinase (SK) inhibitor, does not demonstrate effective anticancer activity in some cancer cells, unlike other known SK1 inhibitors. PF-543 has a non-lipid structure with a unique toluene backbone; however, the importance of this structure remains unclear. Therefore, the purpose of this study was to investigate changes in SK inhibitory and anticancer activities and to explore the role of the tolyl group structure of PF-543 through various modifications. We transformed the methyl group of PF-543 into hydrogen, fluorine, and hydroxy. PF-543 derivatives in which the methyl group was substituted by hydrogen and fluorine (compound **5**) demonstrated SK1 inhibitory and anticancer activities similar to PF-543. Moreover, we performed molecular modeling studies of PF-543 and compound **5**. To assess the metabolic stability of PF-543 and compound **5**, we determined their degree of degradation using the liver microsomes of four different animal species (human, dog, rat, and mouse). However, both PF-543 and compound **5** showed poor microsomal stability. Therefore, for the medical applications of PF-543, the structural modifications of its other parts may be necessary. Our results provide important information for the design of additional PF-543 analogs.

## 1. Introduction

**Figure 1:**
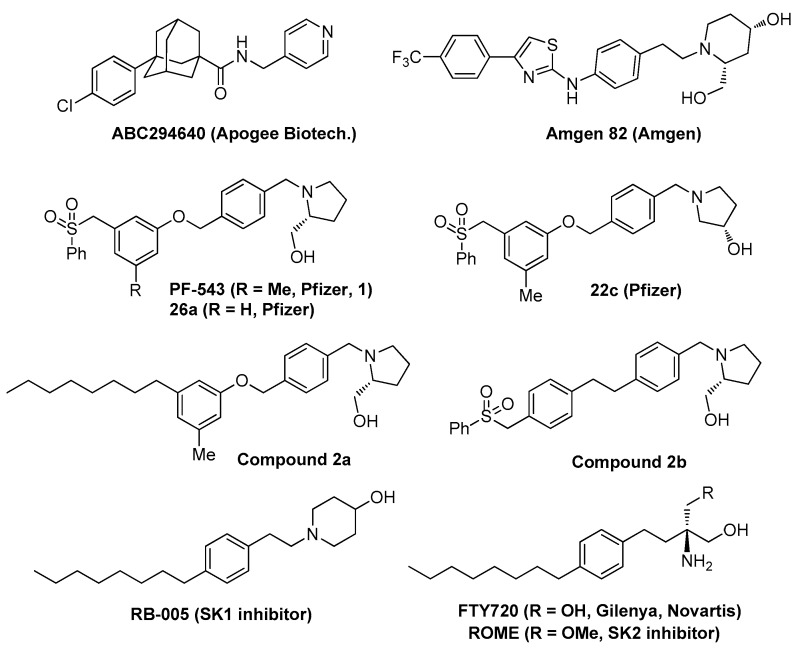
Known sphingosine kinase inhibitors and FTY720.

Sphingolipids are structurally modified by various enzymes and regulate various biological activities through the regulation of cancer cell signaling. Sphingosine and ceramide mediate apoptosis, whereas sphingosine-1-phosphate (S1P), sphingomyelin, and glucosylceramide have antiapoptotic and prosurvival roles. The majority of enzymes involved in sphingolipid metabolism have been identified through comprehensive research, and the regulation of these enzymes has the potential to be developed as a therapeutic agent for various diseases, including cancer [1]. Sphingosine kinase (SK) has two isoforms, i.e., SK1 and SK2, and these play various roles at different positions; thus, the development of selective inhibitors is required. SK transforms sphingosine into S1P, which induces cell growth [1]. In fact, S1P and SK activities increase in various forms of cancers, which can be suppressed through SK inhibition. While the association between SK1 and cancer has been extensively studied, the role of SK2 in cancer remains unclear thus far [2]. Nevertheless, ABC294640 (an SK2 inhibitor developed by Apogee Biotechnology) has been shown to inhibit cell growth in various forms of cancer (Figure 1) [3]. Moreover, in vivo efficacy evaluation has demonstrated the effect that SK2 can have on various solid cancers and hematologic malignancies [4].

ABC294640 successfully completed Phase I clinical trials for various solid cancers, during which rapid and biphasic reductions in plasma levels of S1P were reported [5]. Phase Ib and Phase II clinical trials are underway for cholangiocarcinoma, liver cancer, multiple myeloma, lymphoma and sarcoma using ABC294640 as a target for SK2 [1,6]. These trials show that the ceramide induction or S1P suppression could be used as a new chemotherapy strategy.

Amgen 82, developed by Amgen Inc. (California, CA, USA, Figure 1), as another nonlipid SK inhibitor, inhibits human SK1 and SK2 (IC_50_ = 20 and 100 nM, respectively). Amgen 82 displayed anticancer activities in human melanoma and human breast cancer cell lines [7]. However, many SK inhibitors have a sphingolipid structure. For example, many SK inhibitors of the FTY720 type, such as RB-005 and ROME ((*R*)-FTY720-OMe), have already been classified as sphingolipids (Figure 1) [8]. RB-005 is a sphingosine-based SK1-selective inhibitor (IC_50_ = 3.6 μM) and induced the proteasomal degradation of SK1 in human pulmonary arterial smooth muscle cells [9]. ROME, an SK2 inhibitor (Ki = 16 μM), can reduce MCF-7 breast cancer cell proliferation and prevent cell migration [9].

In 2010, FTY720 (Fingolimod, Gilenya, and Novartis) became the first oral treatment for multiple sclerosis to be approved by the US FDA (Figure 1) [10]. FTY720 also activates protein phosphatase 2A to inhibit cell growth in various forms of cancers (hematologic malignancies and liver, bladder, and colorectal cancers). Additionally, FTY720 inhibits SK1 activity [11]. Diverse in vivo effects of these sphingolipids and FTY720 analogs have been a major obstacle in their development as drugs. Thus, the development of a sphingolipid regulator with a nonlipid structure and selective function is required.

PF-543 [(*R*)-(1-(4-((3-methyl-5-(phenylsulfonyl)phenoxy)methyl)benzyl)pyrrolidin-2-yl)methanol] is a nonlipid SK1 inhibitor (IC_50_ = 2.0 nM) developed by Pfizer Inc. (NYC, NY, USA) and is the most potent SK1 inhibitor to date (Figure 1) [12]. Despite its high SK1 inhibitory effects, PF-543 has been reported to be less effective against certain forms of cancer, including prostate cancer [13]. The assumed reason for this is that PF-543 increases cellular sphingosine levels. Thus, the development of various derivatives of PF-543 is required.

PF-543 has a backbone linked by oxygen and a benzene sulfonyl tail, neither of which exists in other SK inhibitors. Additionally, PF-543 has a unique toluene structure and is generally unstable during in vivo metabolism [14]. Pfizer synthesized a derivative of PF-543, which was modified to have a benzene backbone (**26a**; Figure 1). However, its study revealed that its selective SK1 inhibitory effect was lower than that of PF-543. No additional studies have been conducted on its anticancer activities against compound **26a [15]**. The current knowledge regarding the crystal structures of SK1 and PF-543 make it difficult to confirm whether the toluene structure of PF-543 is needed [16]. We recently reported studies on the PF-543 analogs compound **2a**, which has an aliphatic side chain, and compound **2b**, which has a diphenyl ethane backbone (Figure 1) [17,18]. In both analogs, the inhibitory effect of SK1 was similar to that of PF-543, but the anticancer activities were similar to or slightly improved from PF-543. These results indicate the need for the further investigation of whether the presence of the tolyl group in the PF-543 structure is important for its anticancer activity.

Despite the high SK1 inhibitory effect of PF-543, many analogs have not been developed due to its low anticancer activity. PF-543 analogs reported only SK1/2 inhibitory effects, and based on these results, it is difficult to determine whether anticancer activity improved. Therefore, this study aimed to confirm the effect of the presence of the tolyl group in the PF-543 structure on anticancer activity and to investigate the structural necessity.

## 2. Results and Discussion

### 2.1. Chemical Synthesis

**Scheme 1:**
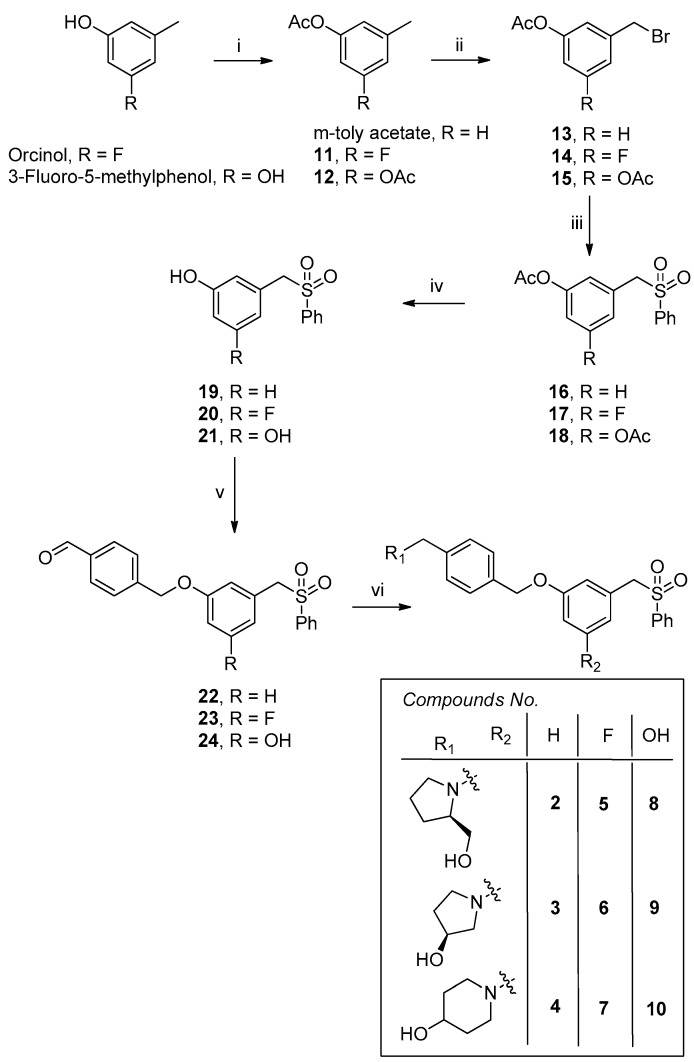
Synthesis of PF-543 derivatives (**2**–**10**). Reagents and conditions: (i) Ac_2_O, pyridine, rt, 12 h; (ii) NBS, EtOAc, 60 °C, 2 d, 52%, (iii) PhSO_2_Na, tetrahydrofuran (THF)/ dimethylformamide (DMF) (2/1), 80 °C, 4 d, 68%; (iv) sat. NaHCO_3_, MeOH, rt, 1 d, 82%; (v) 4-(bromomethyl)benzaldehyde, K_2_CO_3_, THF, 12 h, 62%; (vi) cyclic amines, sodium triacetoxyborohydride (STB), 1,2-dichloroethane, rt, 12 h, 58%–68%.

PF-543 analogs containing fluorine and hydroxyl groups in their modified benzene backbones were synthesized using orcinol and 3-fluoro-5-methylphenol as starting materials, respectively (Scheme 1).

First, compounds **11** and **12** were synthesized by protecting the hydroxyl groups of the starting materials with acetyl. To synthesize PF-543 derivatives that had been modified from toluene to benzene, a reaction was carried out using commercially available *m*-toly acetate. Compounds **11** and **12** and *m*-toly acetate were brominated with *N*-bromosuccinimide, and sulfonyl groups were introduced using benzenesulfonyl sodium salts. After deprotecting the acetyl groups of the obtained compounds (**16**–**18**), compounds **22**–**24** (containing aldehyde) were synthesized using 4-(bromomethyl)benzaldehyde in the same manner as PF-543. Previous results reported by Pfizer revealed slightly elevated SK1 inhibitory effects and selectivity of PF-543 derivative with a hydroxypyrrolidine head group (**22c**; Figure 1) [15]. In addition, some SK inhibitors with an introduced piperidine head group demonstrated high SK1 selectivity and anticancer activity (RB005; Amgen82; Figure 1) [4,8]. These results indicate that the structure of the head group of SK inhibitors is highly important for SK1/2 selectivity and anticancer activity. Therefore, we introduced (*R*)-prolinol, (*S*)-(-)-3-hydroxypyrrolidine and 4-hydroxypiperidine into compounds **22**–**24** using reductive amination. Finally, compounds **2**–**10** with PF-543 containing benzene, fluorobenzene, and phenol were obtained.

### 2.2. SK Activity Assay of PF-543 (1) and Derivatives (**2**–**10**) 

**Figure 2:**
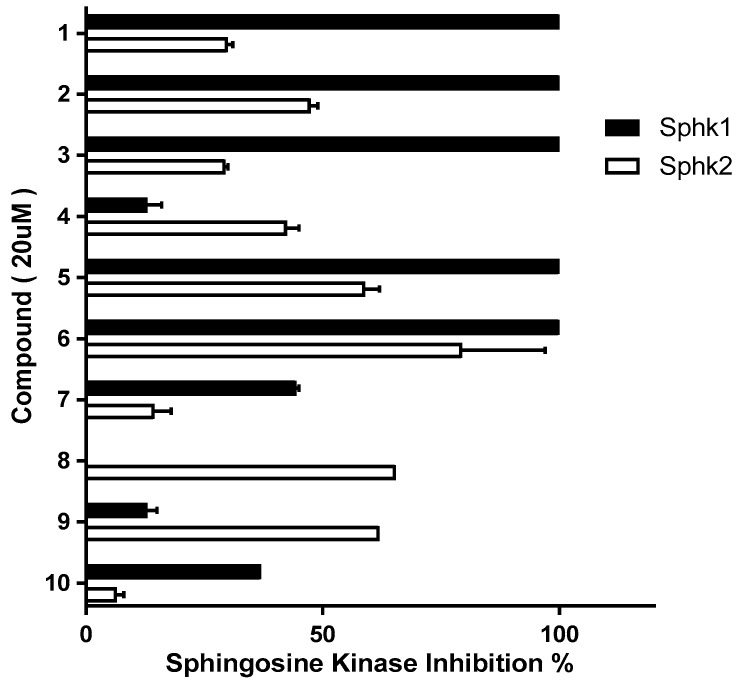
Effect of PF-543 (**1**) and derivatives (**2**–**10**) on SK1/2 activity.

Once synthesized, compounds **2**–**10** were treated with 20 μM using a sphingosine kinase assay kit to measure SK1/2 inhibition (Figure 2). Compounds **8**–**10**, which contained an introduced hydroxyl group, showed a weaker SK1 inhibitory effect. Among compounds **2**–**7** (which contained the introduced hydrogen and fluorine), the SK1 inhibitory effects reduced in compounds **4** and **7**, which also contained an introduced 4-hydroxypiperidine. As a result, it was confirmed that the backbone structure of the polar phenol group replacing methylbenzene in the PF-543 structure reduced SK1 inhibition. Compounds **2** and **5**, which had the same headgroups as PF-543, and compounds **3** and **6**, which had the same headgroups as compound **22c** (Figure 1), inhibited 100% of SK1 similar to PF-543 (at the same concentration). Compounds **4** and **7** had a 4-hydroxypiperidyl headgroup and displayed significantly reduced SK inhibition. This indicates that the pyrrolidine structure is an effective headgroup in PF-543 analogs compared with the piperidine structure. The SK inhibitory effects of compounds **2**, **3**, **5**, and **6** indicated that the presence of the tolyl group in the PF-543 structure did not alter the inhibitory effects of SK.

### 2.3. Cytotoxic Effects of PF-543 (1) and Derivatives (2–10) in Cancer Cells

**Figure 3:**
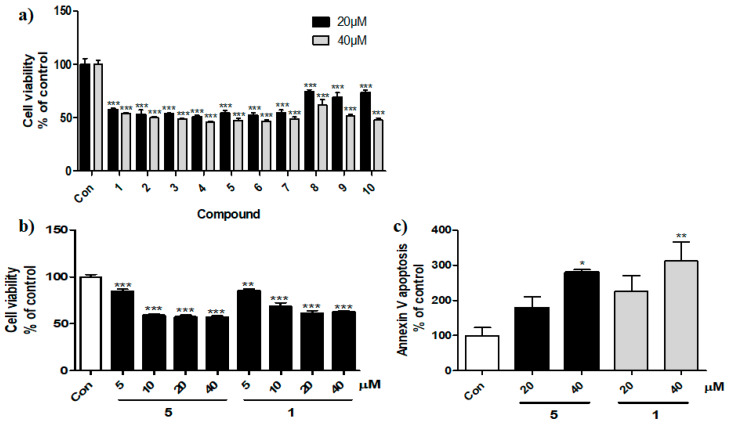
Cytotoxic effects of PF-543 (**1**) and derivatives (**2**–**10**) in cancer cells. (**a**) Cell viability was measured in HT29 cells treated with 20 and 40 μM PF-543 and derivatives. (**b**) Compound **5** and PF-543 were treated in HT29 cells. (**c**) Apoptotic cells were stained with annexin-V-FITC (fluorescein isothiocyanate). * *p* < 0.05, ** *p* < 0.01, *** *p* < 0.001compared with control cells.

The cytotoxicity of compounds **2**–**10** and PF-543 at concentrations of 20 and 40 μM, respectively, was tested against HT29 cancer cells (Figure 3a). The cytotoxic effects of compounds **8**–**10** were weaker than those of PF-543 in colorectal cancer cells, similar to their decreased SK1 inhibitory effects. This indicates that polar groups in the SK1 inhibitor backbones may have reduced SK1 inhibitory and anticancer activities. In contrast, compounds **2**–**7** (including compounds **4** and **7**) demonstrated anticancer activity similar to PF-543 against colorectal cancer cells. Additional studies were conducted on compound **5**, which had the same headgroup structure as PF-543, to confirm the necessity of the tolyl group. Because compound **2**, which also had the same headgroup as PF-543, is a known compound [15], anticancer activities were confirmed using compound **5** introduced with fluorine, which is a bioisostere of hydrogen. An evaluation of compound **5** revealed both SK1 inhibitory activity and cytotoxicity. The cytotoxic effect of compound **5** was similar to that of PF-543. The apoptotic effects of compound **5** and PF-543 were further compared at concentrations of 20 and 40 μM, respectively. It was revealed that compound **5** had similar apoptotic effects. Therefore, the tolyl group in the PF-543 structure did not affect the anticancer activity in the same way as the SK1 inhibitory effects.

### 2.4. Docking Study of PF-543 and Compound **5**

**Figure 4:**
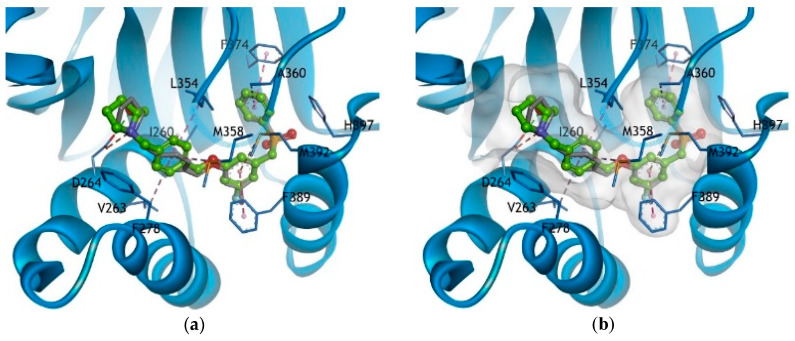
Docking model of **5** (light green, ball and stick model) superimposed with the x-ray binding conformation of PF-543 (gray, stick model) to SK1 (blue ribbon model) (**a**). The hydrogen bond is shown as a green dashed line, and electrostatic interactions are displayed as an orange dashed line. Moreover, the hydrophobic interactions are shown as a pink dashed line and the surface model of the active site bound to **5** is displayed (**b**). For clarity, only the key residues are visible in the stick model and are labeled using the 1-letter amino acid code.

Molecular modeling studies of PF-543 and compound **5** were conducted. The hydroxymethyl-pyrrolidine (OH and N of pyrrolidine) of compound **5** showed both hydrogen bonding and electrostatic interaction with Asp264 (protonated amine form). The phenyl linker of compound **5** showed hydrophobic interactions involving Ile260, Val263, Leu354 and Met358, while the benzene backbone of compound **5** showed hydrophobic interactions with the surrounding Phe389 and Met392. The terminal phenyl group of compound **5**, similar to that of PF-543, formed hydrophobic interactions with aromatic rings and the surrounding Ala360 and Phe374. It was thus determined that compound **5** showed a binding mode similar to PF-543, indicating that its structure can replace the methyl group of PF-543 (Figure 4). 

### 2.5. Metabolic Stability of PF-543 and Compound **5**

To assess the metabolic stability of PF-543 and compound **5**, we determined their degree of degradation using the liver microsomes of four different animal species (human, dog, rat, and mouse). PF-543 and compound **5** both demonstrated a low microsomal stability of <10% in all animal species (Table 1). These results show that the tolyl group of PF-543 did not affect the stability of PF-543.

## 3. Experimental Section

### 3.1. Synthesis in General

The reagents used in the reaction were used by purchasing a commercially available reagent. Column chromatography was performed on silica gel grade 60 (230–400 mesh). All solvents used in the reaction were commercially available anhydrous solvents. ^1^H-NMR and ^13^C-NMR used JEOL ECZ500R (JEOL Co., Tokyo, Japan) and were measured using deuterated solvents at 500 and 125 MHz, respectively. High-resolution mass spectra were measured using an Agilent Technologies G6520A Q-TOF mass spectrometer (Santa Clara, CA, USA) instrument using electrospray ionization (ESI).

### 3.2. Synthesis

#### 3.2.1. 3-Fluoro-5-methylphenyl acetate (**11**)

3-Fluoro-5-methyl phenol (2 g, 0.016 mol) was dissolved in pyridine (80 mL), acetic anhydride (3.0 mL, 0.032 mol) was added, and the mixture was stirred at room temperature for 12 h. Water was added to stop the reaction, and it was concentrated under reduced pressure after EtOAc extraction and MgSO_4_ drying. The mixture was separated by column chromatography (*n*-hexane:EtOAc = 20:1) to give compound **11** (2.64 g, 98%): ^1^H-NMR (500 MHz, CDCl_3_) δ 6.78–6.74 (m, 1H), 6.69 (ddd, *J* = 2.7, 1.4, 0.7 Hz, 1H), 6.66–6.62 (m, 1H), 2.33 (s, 3H), 2.27 (s, 3H); ^13^C-NMR (125 MHz, CDCl_3_) δ 169.3, 163.8, 161.8, 151.3, 151.2, 141.2, 141.1, 118.1, 113.8, 113.6, 106.9, 106.7, 21.5, 21.2; ESI-HRMS (M + H)^+^ *m/z* calcd for C_9_H_10_FO_2_ 169.0665, found 169.0632.

#### 3.2.2. 3-(Bromomethyl)-5-fluorophenyl acetate (**14**)

Compound **11** (1.5 g, 0.0089 mol) was placed in a sealed tube, dissolved in EtOAc (30 mL), and *N*-bromosuccinimide (1.59 g, 0.0089 mol) was added thereto, followed by stirring at 60 °C for 2 days. The reaction was terminated with water and EtOAc, and the organic layer was dried over MgSO_4_ and concentrated under reduced pressure. The resulting mixture was separated by column chromatography (*n*-hexane:EtOAc = 20:1) to give compound **14** (1.03 g, 47%): ^1^H-NMR (500 MHz, CDCl_3_) δ 7.00–6.96 (m, 1H), 6.94–6.93 (m, 1H), 6.80 (dt, *J* = 9.1, 2.2 Hz, 1H), 4.40 (s, 2H), 2.29 (s, 3H); ^13^C-NMR (125 MHz, CDCl_3_) δ 168.9, 163.7, 161.7, 151.6, 151.5, 140.6, 118.2, 118.1, 113.7, 113.5, 109.9, 109.7, 31.7, 31.6, 21.2; ESI-HRMS (M + H)^+^ *m/z* calcd for C_9_H_9_BrFO_2_ 246.9770, found 246.9733. 

#### 3.2.3. 5-(Bromomethyl)-1,3-phenylene diacetate (**15**)

Orcinol (2 g, 0.016 mol) was dissolved in pyridine (80 mL), acetic anhydride (4.57 mL, 0.048 mol) was added thereto, and the mixture was stirred at room temperature for 12 h. Water was added to stop the reaction, and it was concentrated under reduced pressure after EtOAc extraction and MgSO_4_ drying. The resulting mixture **12** (1.7 g, 0.008 mol) was dissolved in EtOAc (50 mL) without purification, and *N*-bromosuccinimide (1.89 g, 0.01 mol) was added thereto and stirred at 60 °C for 2 days. The reaction was terminated with water and EtOAc, and the organic layer was dried over MgSO_4_ and concentrated under reduced pressure. The resulting mixture was separated by column chromatography (*n*-hexane:EtOAc = 5:1) to give compound **15** (1.19 g, 52%): ^1^H-NMR (500 MHz, CDCl_3_) δ 7.02 (d, *J* = 2.1 Hz, 2H), 6.86 (t, *J* = 2.1 Hz, 1H), 4.41 (s, 2H), 2.26 (s, 6H); ^13^C-NMR (125 MHz, CDCl_3_) δ 168.9, 515.1, 139.9, 119.7, 115.5, 31.9, 21.2; ESI-HRMS (M + H)^+^ *m/z* calcd for C_11_H_12_BrO_4_ 286.9919, found 286.9947.

#### 3.2.4. 3-Fluoro-5-((phenylsulfonyl)methyl)phenyl acetate (**17**)

Compound **14** (1.1 g, 0.0045 mol) was placed in a sealed tube, dissolved in THF/DMF (2/1, 30 mL), and benzene sulfinic acid sodium salt (2.2 g, 0.013 mol) was added thereto. The reaction was stirred for 3 days while heating to 80 °C. After the reaction was cooled to room temperature, the reaction was terminated with water and EtOAc, dried over MgSO_4_ and concentrated under reduced pressure. The mixture was separated by column chromatography (*n*-hexane:EtOAc = 2:1) to give compound **17** (1.1 g, 80%): ^1^H-NMR (500 MHz, CDCl_3_) δ 7.69–7.65 (m, 2H), 7.65–7.60 (m, 1H), 7.51–7.46 (m, 2H), 6.84 (t, *J* = 2.2 Hz, 0.5H), 6.82 (t, *J* = 2.2 Hz, 0.5H), 6.71 (t, *J* = 6.71 Hz, 2H), 6.68 (t, *J* = 2.1 Hz, 0.5H), 6.66 (t, *J* = 2.0 Hz, 0.5H), 4.26 (s, 2H), 2.25 (s, 3H); ^13^C-NMR (125 MHz, CDCl_3_) δ 168.8, 163.5, 161.5, 151.5. 151.4, 137.5, 134.2, 131.0, 130.9, 129.3, 128.7, 120.1, 115.5, 115.3, 110.5, 110.3, 62.2, 21.1; ESI-HRMS (M + H)^+^ *m/z* calcd for C_15_H_14_FO_4_S 309.0597, found 309.0543.

#### 3.2.5. 5-((Phenylsulfonyl)methyl)-1,3-phenylene diacetate (**18**)

Compound **15** (0.9 g, 0.0031 mol) was dissolved in 25 mL of THF/DMF (2/1) mixed solvent in a sealed tube, benzenesulfinic acid sodium salt (777 mg, 0.0047 mol) was added, and the mixture was stirred at 80 °C for 4 days. The reaction was terminated with water and EtOAc, dried over MgSO_4_ and concentrated under reduced pressure. The mixture was separated by column chromatography (*n*-hexane:EtOAc = 3:1) to give compound **18** (744 mg, 68%): ^1^H-NMR (500 MHz, CDCl_3_) δ 7.53–7.61 (m, 2H), 7.59–7.56 (m, 1H), 7.44–7.43 (m, 2H), 6.87 (t, *J* = 2.1 Hz, 1H), 6.73 (d, *J* = 2.1 Hz, 2H), 4.24 (s, 2H), 2.21 (s, 6H); ^13^C-NMR (125 MHz, CDCl_3_) δ 168.5, 150.8, 137.3, 133.7, 128.9, 128.4, 121.2, 115.8, 61.9, 20.8; ESI-HRMS (M + H)^+^ *m/z* calcd for C_17_H_17_O_6_S 349.0746, found 349.0721.

#### 3.2.6. 3-((Phenylsulfonyl)methyl)phenol (**19**)

*m*-Toly acetate (2 g, 0.013 mol) was dissolved in EtOAc (150 mL), and *N*-bromosuccinimide (2.37 g, 0.0133 mol) was added thereto, followed by stirring for 3 days while heating to 70 °C. After cooling to room temperature, water and EtOAc were added thereto and the reaction was completed. The organic layer was separated, dried over MgSO_4_, and concentrated to obtain a mixture of compound **13**. Compound **13** was placed in a sealed tube and dissolved in 120 mL of THF/DMF (2/1). Benzenesulfinic acid sodium salt (4.27 g, 0.026 mol) was added thereto, followed by stirring for 3 days while heating to 80 ^o^C. The reaction was cooled to room temperature, water and EtOAc were added and the reaction was completed. The organic layer was separated, dried over MgSO_4_, and concentrated to obtain mixture compound **18**. The reaction mixture was dissolved in 150 mL of MeOH/H_2_O (1/1), NaHCO_3_ (5 g) was added thereto, and the mixture was stirred for 12 h while heating at 40 °C. The reaction was cooled to room temperature, water and EtOAc were added thereto and the reaction was completed. The organic layer was separated, dried over MgSO_4_, and concentrated. The mixture was separated by column chromatogrphy (*n*-hexane:EtOAc = 2:1) to obtain compound **19**. The chemical spectrum of compound **19** was consistent with that previously reported [15].

#### 3.2.7. 3-Fluoro-5-((phenylsulfonyl)methyl)phenol (**20**)

Compound **17** (1.0 g, 0.0032 mol) was dissolved in MeOH/H_2_O (1/1, 60 mL) and NaHCO_3_ (2 g) was added thereto. The reaction was stirred for 1 day while heating to 40 °C. The reaction was terminated with water and EtOAc, dried over MgSO_4_ and concentrated under reduced pressure. The mixture was separated by column chromatography (CH_2_Cl_2_:MeOH = 20:1) to give compound **20** (783 mg, 92%): ^1^H-NMR (500 MHz, CDCl_3_) δ 7.66 (dd, *J* = 5.2, 3.4 Hz, 2H), 7.62 – 7.57 (m, 1H), 7.46 (t, *J* = 7.8 Hz, 2H), 6.53 (t, *J* = 2.3 Hz, 0.5 H), 6.51 (t, *J* = 2.2 Hz, 0.5 H), 6.50 (t, *J* = 1.7 Hz, 1H), 6.25–6.23 (m, 0.5H), 6.23–6.21 (m, 0.5H), 4.21 (s, 2H); ^13^C-NMR (125 MHz, CDCl_3_) δ 164.3, 163.4, 162.3, 158.1, 158.0, 137.5, 134.2, 130.4, 130.3, 129.3, 129.2, 128.6, 114.1, 109.5, 109.4, 104.0, 103.8, 62.5; ESI-HRMS (M + H)^+^ *m/z* calcd for C_13_H_12_FO_3_S 267.0491, found 267.0466.

#### 3.2.8. 5-((Phenylsulfonyl)methyl)benzene-1,3-diol (**21**)

Compound **18** (700 mg, 0.002 mol) was dissolved in MeOH (25 mL) and aq. NaHCO_3_ (10 mL) was added thereto. It was stirred at room temperature for 1 day. The reaction was terminated with water and EtOAc, dried over MgSO_4_ and concentrated under reduced pressure. The mixture was separated by column chromatography (CH_2_Cl_2_:MeOH = 10:1) to give compound **21** (436 mg, 82%): ^1^H NMR (500 MHz, CDCl_3_) δ 7.68 (dd, *J* = 8.4, 1.2 Hz, 2H), 7.62–7.57 (m, 1H), 7.49–7.41 (m, 2H), 6.30 (t, *J* = 2.2 Hz, 1H), 6.15 (d, *J* = 2.2 Hz, 2H), 5.28 (s, 2H); ^13^C-NMR (125 MHz, CDCl_3_) δ 158.0, 137.7, 133.9, 129.4, 129.0, 128.5, 109.5, 103.2, 62.6; ESI-HRMS (M + H)^+^ *m/z* calcd for C_13_H_13_O_4_S 265.0535, found 265.0581.

#### 3.2.9. 4-((3-((Phenylsulfonyl)methyl)phenoxy)methyl)benzaldehyde (**22**)

Compound **19** (292 mg, 1.176 mmol) was dissolved in MeCN (10 mL), and K_2_CO_3_ (488 mg, 3.528 mmol) and 4-(bromomethyl)benzaldehyde (257 mg, 1.294 mmol) were added thereto, and the mixture was stirred for 12 h while heating to 60 °C. After cooling the reaction to room temperature, the reaction was terminated with water and EtOAc, dried over MgSO_4_ and concentrated under reduced pressure. The reaction was washed with *n*-hexane/EtOAc (3/1) to give compound **22**. The chemical spectrum of compound **22** was consistent with that previously reported [15].

#### 3.2.10. 4-((3-Fluoro-5-((phenylsulfonyl)methyl)phenoxy)methyl)benzaldehyde (**23**)

Compound **20** (0.7 g, 0.0026 mol) was dissolved in MeCN (30 mL), and K_2_CO_3_ (1.09 g, 0.079 mol) and 4-(bromomethyl)benzaldehyde (569 mg, 0.0029 mmol) were added thereto. The reaction was stirred at room temperature for 12 h, the reaction was terminated with water and EtOAc, dried over MgSO_4_ and concentrated under reduced pressure. The mixture was washed with (*n*-hexane:EtOAc = 3:1) to give compound **23** (730 mg, 73%): ^1^H-NMR (500 MHz, CDCl_3_) δ 10.00 (s, 1H), 7.88 (d, *J* = 8.2 Hz, 2H), 7.66 (dd, *J* = 8.4, 1.2 Hz, 2H), 7.64–7.59 (m, 1H), 7.52 (d, *J* = 8.1 Hz, 2H), 7.49–7.43 (m, 2H), 6.63 (t, *J* = 2.3 Hz, 0.5H), 6.61 (t, *J* = 2.3 Hz, 0.5H), 6.56 (t, *J* = 1.7 Hz, 1H), 6.41–6.40 (m, 0.5H), 6.39–6.38 (m, 0.5H), 5.03 (s, 2H), 4.23 (s, 2H); ^13^C-NMR (125 MHz, CDCl_3_) δ 191.9, 164.2, 162.3, 159.6, 159.5, 143.0, 137.8, 136.2, 134.1, 131.0, 130.2, 129.2, 128.6, 127.6, 113.1, 110.9, 110.8, 103.5, 103.3, 69.6, 62.5; ESI-HRMS (M + H)^+^ *m/z* calcd for C_21_H_18_FO_4_S 385.0910, found 385.0981.

#### 3.2.11. 4-((3-Hydroxy-5-((phenylsulfonyl)methyl)phenoxy)methyl)benzaldehyde (**24**)

Compound **21** (310 mg, 1.173 mmol) was dissolved in THF (15 mL), and K_2_CO_3_ (650 mg, 4.70 mmol) and 4-(bromomethyl)benzaldehyde (210 mg, 1.056 mmol) were added thereto. The reaction was stirred at room temperature for 12 h and terminated with water and EtOAc. Then, it was dried over MgSO_4_ and concentrated under reduced pressure. The mixture was separated by column chromatography (CH_2_Cl_2_:MeOH = 20:1) to give compound **24** (278 mg, 62%): ^1^H-NMR (500 MHz, CDCl_3_) δ 9.93 (s, 1H), 7.84 (d, *J* = 8.0 Hz, 2H), 7.64 – 7.60 (m, 2H), 7.58 (d, *J* = 7.3 Hz, 1H), 7.50 (d, *J* = 8.0 Hz, 2H), 7.45 (d, *J* = 7.7 Hz, 2H), 6.36 (t, *J* = 2.2 Hz, 1H), 6.17 (t, *J* = 1.6 Hz, 1H), 6.15 (t, *J* = 1.6 Hz, 1H), 4.96 (s, 2H), 4.20 (s, 2H); ^13^C-NMR (125 MHz, CDCl_3_) δ 192.7, 159.5, 158.3, 144.2, 137.7, 135.8, 133.9, 130.0, 129.0, 128.5, 127.5, 111.2, 108.5, 102.9, 69.1, 62.7; ESI-HRMS (M + H)^+^ *m/z* calcd for C_21_H_19_O_5_S 383.0953, found 383.0914.

#### 3.2.12. (R)-(1-(4-((3-((Phenylsulfonyl)methyl)phenoxy)methyl)benzyl)pyrrolidin-2-yl)methanol (**2**) 

Compound **22** (63 mg, 0.17 mmol) was dissolved in 1,2-dicholroethane (5 mL), and (*R*)-(-)-prolinol (52 mg, 0.516 mmol) and sodium triacetoxyborohydride (73 mg, 0.34 mmol) were added thereto. The mixture was stirred for 12 h at room temperature. The reaction was terminated with water and EtOAc, dried over MgSO_4_ and concentrated under reduced pressure. The mixture was separated by column chromatography (CH_2_Cl_2_:MeOH = 5:1) to give compound **2** (46 mg, 60%) [15]: ^1^H-NMR (500 MHz, CDCl_3_) δ 7.63 (dd, *J* = 8.4, 1.2 Hz, 2H), 7.61–7.57 (m, 1H), 7.45 (d, *J* = 7.6 Hz, 2H), 7.43 (d, *J* = 7.6 Hz, 2H), 7.36 (d, *J* = 8.1 Hz, 2H), 7.15–7.10 (m, 1H), 6.89 (ddd, *J* = 8.3, 2.5, 0.8 Hz, 1H), 6.74–6.70 (m, 1H), 6.62 (d, *J* = 7.6 Hz, 1H), 4.93 (s, 2H), 4.25 (s, 2H), 4.14 (d, *J* = 13.1 Hz, 1H), 3.70 (dt, *J* = 16.6, 8.3 Hz, 2H), 3.58 (dd, *J* = 11.8, 3.9 Hz, 1H), 3.18–3.16 (m, 1H), 3.11–3.01 (m, 1H), 2.53 (dd, *J* = 16.7, 8.6 Hz, 1H), 1.97 (ddd, *J* = 15.8, 9.6, 5.8 Hz, 2H), 1.84 (ddtd, *J* = 15.8, 12.3, 8.4, 4.6 Hz, 4H); ^13^C-NMR (125 MHz, CDCl_3_) δ 158.7, 138.0, 133.9, 130.0, 129.7, 129.5, 129.0, 128.7, 127.9, 123.6, 117.0, 115.8, 69.6, 62.9, 61.5, 58.6, 54.3, 27.2, 23.5; ESI-HRMS (M + H)^+^ *m/z* calcd for C_26_H_30_NO_4_S 452.1896, found 452.1877.

#### 3.2.13. (S)-1-(4-((3-((Phenylsulfonyl)methyl)phenoxy)methyl)benzyl)pyrrolidin-3-ol (**3**)

Using compound **22** (63 mg, 0.17 mmol) and (*S*)-(-)-3-hydroxy pyrrolidine (45 mg, 0.516 mmol), compound **3** (38 mg, 52%) was obtained by the same method as the synthesis method of compound **2**: ^1^H-NMR (500 MHz, CDCl_3_) δ 7.63 (dd, *J* = 8.4, 1.2 Hz, 2H), 7.61–7.57 (m, 1H), 7.46–7.44 (m, 2H), 7.45–7.42 (m, 2H), 7.16–7.09 (m, 1H), 6.92–6.85 (m, 1H), 6.74–6.70 (m, 1H), 6.61 (d, *J* = 7.6 Hz, 1H), 4.92 (s, 2H), 4.40–4.39 (m, 1H), 4.25 (s, 2H), 3.90 (d, *J* = 7.9 Hz, 2H), 3.17–3.07 (m, 1H), 2.95 (d, *J* = 11.1 Hz, 1H), 2.88 (dd, *J* = 11.1, 5.0 Hz, 1H), 2.72 (dd, *J* = 15.2, 9.2 Hz, 1H), 2.25–2.13 (m, 1H), 1.95–1.82 (m, 1H); ^13^C-NMR (125 MHz, CDCl_3_) δ 158.7, 137.9, 137.0, 133.9, 130.0, 129.7, 129.4, 129.2, 128.7, 127.9, 123.7, 117.1, 115.7, 70.5, 69.6, 62.9, 62.0, 59.5, 52.4, 34.3; ESI-HRMS (M + H)^+^ *m/z* calcd for C_25_H_28_NO_4_S 438.1739, found 438.1702.

#### 3.2.14. 1-(4-((3-((Phenylsulfonyl)methyl)phenoxy)methyl)benzyl)piperidin-4-ol (**4**)

Using compound **22** (63 mg, 0.17 mmol) and 4-hydroxypiperidine (52 mg, 0.516 mmol), compound **4** (53 mg, 69%) was obtained by the same method as the synthesis of compound **2**: ^1^H-NMR (500 MHz, CDCl_3_) δ 7.63–7.60 (m, 2H), 7.58 (dd, *J* = 10.6, 4.4 Hz, 1H), 7.43 (dd, *J* = 8.1, 7.6 Hz, 2H), 7.40 (d, *J* = 7.9 Hz, 2H), 7.34 (d, *J* = 8.0 Hz, 2H), 7.12 (t, *J* = 7.9 Hz, 1H), 6.94–6.87 (m, 1H), 6.75–6.69 (m, 1H), 6.60 (d, *J* = 7.6 Hz, 1H), 4.92 (s, 2H), 4.25 (s, 2H), 3.77–3.74 (m, 1H), 3.68 (s, 2H), 2.94–2.82 (m, 1H), 2.57–2.44 (m, 2H), 2.03–1.90 (m, 2H), 1.71–1.57 (m, 2H); ^13^C-NMR (125 MHz, CDCl_3_) δ 158.7, 137.9, 136.5, 133.9, 130.2, 129.7, 129.5, 129.1, 128.8, 127.7, 123.6, 117.0, 115.8, 69.7, 62.9, 61.9, 50.7, 33.0; ESI-HRMS (M + H)^+^ *m/z* calcd for C_26_H_30_NO_4_S 452.1896, found 452.1890.

#### 3.2.15. (R)-(1-(4-((3-Fluoro-5-((phenylsulfonyl)methyl)phenoxy)methyl)benzyl)pyrrolidin-2-yl)methanol (**5**)

Using compound **23** (84 mg, 0.22 mmol) and (*R*)-(-)-prolinol (66 mg, 0.656 mmol), compound **5** (72 mg, 70%) was obtained by the same method as the synthesis of compound **2**: ^1^H-NMR (500 MHz, CDCl_3_) δ 7.65 (dd, *J* = 8.3, 1.2 Hz, 2H), 7.64–7.59 (m, 1H), 7.51 (d, *J* = 8.0 Hz, 2H), 7.49–7.44 (m, 2H), 7.36 (d, *J* = 8.0 Hz, 2H), 6.60 (dt, *J* = 10.4, 2.3 Hz, 1H), 6.52–6.51 (m, 1H), 6.40–6.34 (m, 1H), 4.92 (s, 2H), 4.29 (d, *J* = 13.1 Hz, 1H), 4.21 (s, 2H), 3.90 (d, *J* = 13.1 Hz, 1H), 3.81–3.66 (m, 2H), 3.33–3.22 (m,2H), 2.74–2.66 (m, 1H), 2.04–1.79 (m, 4H); ^13^C-NMR (125 MHz, CDCl_3_) δ 164.2, 162.2, 159.8, 137.8, 137.0, 134.1, 130.8, 129.2, 128.6, 127.9, 113.1, 110.6, 110.5, 103.4, 103.2, 69.8, 67.4, 62.5, 61.3, 58.7, 54.0, 53.6, 50.7, 26.8, 23.4; ESI-HRMS (M + H)^+^ *m/z* calcd for C_26_H_29_FNO_4_S 470.1801, found 470.1819.

#### 3.2.16. (S)-1-(4-((3-fluoro-5-((phenylsulfonyl)methyl)phenoxy)methyl)benzyl)pyrrolidin-3-ol (**6**)

Using compound **23** (65 mg, 0.17 mmol) and (*S*)-(-)-3-hydroxy pyrrolidine (44 mg, 0.51 mmol), compound **6** (43 mg, 56%) was obtained by the same method as the synthesis of compound **2**: ^1^H-NMR (500 MHz, CDCl_3_) δ 7.68–7.64 (m, 2H), 7.64–7.60 (m, 1H), 7.50–7.45 (m, 2H), 7.40 (d, *J* = 8.0 Hz, 2H), 7.34 (d, *J* = 8.1 Hz, 2H), 6.62 (dt, *J* = 10.4, 2.3 Hz, 1H), 6.52–6.51 (m, 1H), 6.40–6.35 (m, 1H), 4.92 (s, 2H), 4.22 (s, 2H), 4.08 (d, *J* = 13.1 Hz, 1H), 3.69 (dd, *J* = 11.5, 3.3 Hz, 1H), 3.61–3.49 (m, 2H), 3.15–3.06 (m, 1H), 2.97–2. 92 (m, 1H), 2.44 (dt, *J* = 15.7, 8.0 Hz, 1H), 2.02–1.82 (m, 2H); ^13^C-NMR (125 MHz, CDCl_3_) δ 164.2, 162.3, 159.9, 137.8, 134.1, 130.8, 129.8, 129.2, 128.7, 127.8, 113.1, 110.6, 110.4, 103.5, 103.3, 70.1, 62.6, 61.6, 58.5, 54.4, 31.0; ESI-HRMS (M + H)^+^ *m/z* calcd for C_25_H_27_FNO_4_S 456.1645, found 456.1697.

#### 3.2.17. 1-(4-((3-Fluoro-5-((phenylsulfonyl)methyl)phenoxy)methyl)benzyl)piperidin-4-ol (**7**)

Using compound **23** (65 mg, 0.17 mmol) and 4-hydroxypiperidine (51 mg, 0.51 mmol), compound **7** (56 mg, 71%) was obtained by the same method as the synthesis of compound **2**: ^1^H-NMR (500 MHz, CDCl_3_) δ 7.66 (dd, *J* = 8.4, 1.2 Hz, 2H), 7.62 (ddt, *J* = 8.7, 7.4, 1.2 Hz, 1H), 7.49–7.45 (m, 2H), 7.36 (d, *J* = 8.0 Hz, 2H), 7.31 (d, *J* = 8.1 Hz, 2H), 6.62 (dt, *J* = 10.4, 2.3 Hz, 1H), 6.54–6.51 (m, 1H), 6.39–6.34 (m, 1H), 4.91 (s, 2H), 4.22 (s, 2H), 3.79–3.65 (m, 1H), 3.55 (s, 2H), 2.86–2.70 (m, 2H), 2.31–2.17 (m, 2H), 1.94–1.86 (m, 2H), 1.65–1.56 (m, 2H); ^13^C-NMR (125 MHz, CDCl_3_) δ 164.2, 162.3, 160.0, 137.8, 134.1, 130.8, 129.7, 129.2, 128.7, 127.6, 113.1, 110.5, 110.3, 103.4, 103.2, 70.2, 62.7, 53.5, 50.9, 34.1, 31.0; ESI-HRMS (M + H)^+^ *m/z* calcd for C_26_H_29_FNO_4_S 470.1801, found 470.1840.

#### 3.2.18. (R)-3-((4-((2-(Hydroxymethyl)pyrrolidin-1-yl)methyl)benzyl)oxy)-5-((phenylsulfonyl)methyl)phenol (**8**)

Compound **24** (58 mg, 0.15 mmol) was dissolved in 1,2-dichloroethane (5 mL), and (*R*)-(-)-prolinol (46 mg, 0.45 mmol) and sodium triacetoxyborohydride (64 mg, 0.303 mmol) were added. It was stirred at room temperature for 12 h. The reaction was terminated with water and EtOAc, dried over MgSO_4_ and concentrated under reduced pressure. The mixture was separated by column chromatography (CH_2_Cl_2_:MeOH = 5:1) to give compound **8** (41 mg, 58%): ^1^H-NMR (500 MHz, CDCl_3_/CD_3_OD = 3/1) δ 7.52–7.48 (m, 2H), 7.46 (td, *J* = 2.0, 1 Hz), 7.35–7.30 (m, 2H), 7.21 (d, *J* = 8.0 Hz, 2H), 7.18 (d, *J* = 8.0 Hz, 2H), 6.23 (t, *J* = 2.2 Hz. 1H), 6.08–6.02 (m, 1H), 6.01–5.98 (m, 1H), 4.72 (s, 2H), 3.17 (d, *J* = 1.6 Hz, 2H), 2.92 (dd, *J* = 5.7, 2.9 Hz, 1H), 2.83 (d, *J* = 3.3 Hz, 1H), 2.37 (dd, *J* = 16.7, 9.1 Hz, 1H), 1.91–1.76 (m, 2H), 1.70–1.55 (m, 4H); ^13^C-NMR (125 MHz, CDCl_3_/CD_3_OD = 3/1) δ 159.8, 158.2, 137.7, 133.9, 129.9, 129.7, 129.0, 128.5, 127.7, 111.0, 108.6, 103.0, 69.6, 65.7, 62.8, 62.1, 58.6, 54.3, 29.6, 27.3, 22.8.; ESI-HRMS (M + H)^+^ *m/z* calcd for C_26_H_30_NO_5_S 468.1845, found 468.1849.

#### 3.2.19. (S)-1-(4-((3-Hydroxy-5-((phenylsulfonyl)methyl)phenoxy)methyl)benzyl)pyrrolidin-3-ol (**9**)

Compound **24** (58 mg, 0.15 mmol) was dissolved in 1,2-dichloroethane (5 mL), and then (*S*)-(-)-3-hydroxypyrrolidine (40 mg, 0.45 mmol) and sodium triacetoxyborohydride (64 mg, 0.455 mmol) were added. It was stirred at room temperature for 12 h. The reaction was terminated with water and EtOAc, dried over MgSO_4_ and concentrated under reduced pressure. The mixture was separated by column chromatography (CH_2_Cl_2_:MeOH = 5:1) to give compound **9** (44 mg, 64%): ^1^H-NMR (500 MHz, CDCl_3_/CD_3_OD = 3/1) δ 7.49 (dd, *J* = 5.9, 4.7 Hz, 2H), 7.46 (d, *J* = 7.5 Hz, 1H), 7.32 (t, *J* = 7.8 Hz, 2H), 7.28 (d, *J* = 8.1 Hz, 2H), 7.21 (d, *J* = 8.1 Hz, 2H), 6.23 (t, *J* = 2.1 Hz, 1H), 6.03 (d, *J* = 1.6 Hz, 1H), 6.01 (d, *J* = 1.7 Hz, 1H), 4.72 (s, 2H), 4.36–4.20 (m, 1H), 4.05 (s, 2H), 3.01–2.91 (m, 2H), 2.86–2.78 (m, 1H), 2.73 (d, *J* = 11.6 Hz, 1H), 2.12 – 1.95 (m, 1H), 1.78–1.64 (m, 1H); ^13^C-NMR (125 MHz, CDCl_3_/CD_3_OD = 3/1) δ 159.7, 158.2, 137.6(2C), 133.9, 130.0, 129.7, 129.0(2C), 128.5, 127.9, 111.0, 108.6, 103.0, 69.7, 69.4, 62.7, 61.4, 59.7, 52.4, 33.7; ESI-HRMS (M + H)^+^ *m/z* calcd for C_25_H_28_NO_5_S 454.1688, found 454.1673.

#### 3.2.20. 1-(4-((3-Hydroxy-5-((phenylsulfonyl)methyl)phenoxy)methyl)benzyl)piperidin-4-ol (**10**)

Compound **24** (58 mg, 0.15 mmol) was dissolved in 1,2-dichloroethane (5 mL), 4-hydroxypiperidine (46 mg, 0.45 mmol) and sodium triacetoxyborohydride (64 mg, 0.455 mmol) were added and stirred at room temperature for 12 h. The reaction was terminated with water and EtOAc, dried over MgSO_4_ and concentrated under reduced pressure. The mixture was separated by column chromatography (CH_2_Cl_2_:MeOH = 5:1) to give compound **10** (48 mg, 68%): ^1^H-NMR (500 MHz, CDCl_3_/CD_3_OD = 3/1) δ 7.54–7.48 (m, 2H), 7.48–7.44 (m, 1H), 7.33 (dd, *J* = 7.0, 1.2 Hz, 2H), 7.26 (d, *J* = 8.1 Hz, 2H), 7.21 (d, *J* = 8.1 Hz, 2H), 6.23 (t, *J* = 2.2 Hz, 1H), 6.03 (d, *J* = 1.6 Hz, 1H), 6.01 (d, *J* = 1.6 Hz, 1H), 4.73 (s, 2H), 4.05 (s, 2H), 3.63–3.65 (m, 1H), 2.85 (ddd, *J* = 11.2, 7.8, 3.3 Hz, 2H), 2.47 (d, *J* = 13.7 Hz, 2H), 1.82 (dd, *J* = 14.8, 6.0 Hz, 2H), 1.67 – 1.62 (m, 2H); ^13^C NMR (125 MHz, CDCl_3_/CD_3_OD = 3/1) δ 159.9, 158.4, 137.8, 134.1, 130.8, 129.8, 129.2, 128.7, 127.9, 111.2, 108.8, 103.2, 69.6, 62.9, 61.4, 53.9, 41.5, 31.8, 29.8; ESI-HRMS (M + H)^+^ *m/z* calcd for C_26_H_30_NO_5_S 468.1845, found 468.1829.

### 3.3. Sphingosine Kinase Activity Assay

SK 1/2 activity was measured using 20 μM PF-543 (**1**) and compounds **2**–**10** using an Adapta^TM^ screening system (Thermo Fisher Scientific system, Waltham, MA, USA). The sphingosine kinase 1 activity assay used 0.04–0.16 ng SPHK1, 50 μM sphingosine lipid substrate in 32.5 mM HEPES pH 7.5, 0.005% BRIJ 35, 5 mM MgCl_2_, 0.5 mM ethylene glycol-bis(β-aminoethyl ether)-*N*,*N*,*N*′,*N*′-tetraacetic acid (EGTA). The sphingosine kinase 2 activity assay detected 35 - 140 ng SPHK2, 50 μM sphingosine lipid substrate in 32.5 mM HEPES pH 7.5, 0.5 mM EGTA, 1.5 mM MgCl_2_. 

### 3.4. Cell Culture and Proliferation Assays

HT29 cells (Korean Cell Line Bank, Seoul, Korea) were maintained in RPMI (Biowest, Nuaillé, France) media with 10% fetal bovine serum (FBS), 100 U/mL penicillin and 100 µg/mL streptomycin at 37 °C in a humidified 5% CO_2_/95% air atmosphere. Cells were seeded in 96-well plates at a density of 3000 cells/100 μL/well and incubated for 24 h. The cells were then incubated in culture medium containing synthetic compounds. Following 24 h of incubation, the cell viability was determined using a EZ-CYTOX kit (DaeilLab Service, Seoul, Korea) according to the manufacturer’s protocol (*n* = 12)**.**

### 3.5. Annexin-V Staining of PF-543 and Derivative **5**

The cell apoptosis assay was determined using annexin V- fluorescein isothiocyanate (FITC) (Biobud, Gyeonggi-do, Korea) and propedium iodide (PI) staining as described in the manufacturer’s instructions (BioBud, Gyeonggi-do, Korea). Briefly, the cells were resuspended in binding buffer and then incubated with annexin-V-FITC and propidium iodide at room temperature. After incubation, stained cells were analyzed by MACSQuant Analyzer 10 Fow Cytometer (Miltenyi Biotec, Bergisch Gladbach, Germany). The experiment was conducted three times independently (*n* = 6).

### 3.6. Molecular Modeling of PF-543 Derivative 5 Against SK1

Molecular modeling study of **5** against the SphK1 was performed using Schrödinger Suite 2019-1 (Schrödinger, LLC, New York, NY, USA, 2019). The PF-543-bound crystal structure of SphK1 was obtained from the Protein Data Bank (http://www.rcsb.org/pdb), the PDB code for which was 4V24 [16]. The protein preparation was revised using Protein Preparation Wizard in Maestro v11.9 (Schrödinger, NYC, NY, USA), and the receptor grid box was generated using a 25 × 25 × 25 Å cubic size centered on complexed ligand. The ligand was minimized using an OPLS3e force field with a dielectric constant value 80.0 in MacroModel v12.3 (Schrödinger, NYC, NY, USA). The ligand docking was performed using the Glide v8.2 program (Schrödinger, NYC, NY, USA) with the Standard Precision method. The figure of the proposed binding mode of C3 was created by Discovery Studio 2018 (Dassault Systèmes, San Diego, CA, USA, 2018).

### 3.7. In Vitro Metabolic Stability of PF-543 and Derivative **5**

Microsomal incubations were conducted in triplicate in 0.1 M potassium phosphate buffer (pH 7.4) in a clean 1.5 mL eppendorf tube. To evaluate reduced nicotinamide adenine dinucleotide phosphate (NADPH)-dependent metabolism, PF-543, derivative **5** or Verapamil (positive control) was incubated with pooled liver microsomes from human (HLM), dog (DLM), rat (RLM) and mouse (MLM) in the presence of an NADPH regenerating system in a final volume of 100 mL. The final incubation mixtures contained 0.5 mg/mL HLM, 0.1 M potassium phosphate buffer (pH 7.4), and an NADPH regenerating system (1 mM NADPH, 10 mM MgCl_2_). HLM was added and the mixture was pre-incubated at 37 °C in a shaking incubator at approximately 350 rpm for 5 min under Thermo mixer (Eppendorf., Hamburg, Germany). Reactions were initiated by the addition of 1 mM NADPH and were then quenched by adding 40 µL of ice-cold acetonitrile containing 10 µM chlorproamide (CPP) as an internal standard at 0, 30 min. The incubation mixtures were then centrifuged at 15,000× *g* for 5 min at 4 °C. A 2 µL aliquot of the supernatant was directly injected into the LC-MS/MS system. The LC-MS/MS system consisted of the Nexera XR HPLC system (Shimadzu Co., Kyoto, Japan) coupled to the TSQ Vantage triple quadrupole mass spectrometer equipped with Xcalibur version 1.1.1 (Thermo Fisher Scientific Inc., Waltham, MA, USA).

### 3.8. Statistical Analysis 

The data were presented as means ± SD. Non–parametric one–way ANOVA was applied to the data with heterogeneous variance. Turkey’s post-procedures were conducted to determine intergroup variation. The difference was considered significant if *p* < 0.05. Graph Pad Prizm 7 (GraphPad Sofrware, Inc., La Jolla, CA, USA) was used to analyze all the data.

## 4. Conclusions

In this study, we synthesized the derivatives of the tolyl group of PF-543 with benzene, fluorobenzene, and phenol to improve the anticancer activity of PF-543. In addition, prolinol, hydroxypyrrolidine, and piperidine were introduced into the headgroups of each backbone derivative, and the SK1 inhibitory and anticancer activities of these synthesized compounds were measured. The inhibitory effects of SK in compounds **4**, **7**, and **10** reduced when a piperidine headgroup was introduced compared with those of PF-543 (**1**). All derivatives with a phenol backbone (compounds **8**–**10**) displayed reduced SK1 inhibitory effects. Only compound **8** showed an inhibitory effect against SK2 alone. These results indicate that the tolyl group of PF-543 affects its SK inhibitory effect. In contrast, compounds with a benzene and fluorobenzene backbone (**2**–**7**) demonstrated anticancer activity similar to PF-543. In addition, the fluorine-substituted compound **5**, with the same headgroup as PF-543, showed similar apoptotic effects. The molecular docking of PF-543 and compound **5** did not reveal any difference in binding between the two compounds. However, their microsomal stability was low in all tested animal species. Collectively, our results indicate that, although the fluorobenzene backbone can replace the PF-543 backbone, it does not improve its stability.

Despite the strong SK1 inhibitory effects of PF-543, various derivatives have not been developed due to its low anticancer activity and structural instability. The PF-543 analogs reported by Pfizer and other research groups provided only comparative information on the inhibitory effects of SK1/2; therefore, it remains unknown if the derivatives improved anticancer activity. The purpose of this study was to determine if the presence of the tolyl group in the PF-543 structure is required for its anticancer activity. Our results show that the tolyl group did not significantly affect the SK1 inhibitory effects and anticancer activities of PF-543. Furthermore, synthesized PF-543 derivative did not create additional structural stability. These results showed that it is possible to design a simplified PF-543 analog in which the tolyl group has been removed. The structural stability of PF-543 could be improved by designing derivatives in which a functional group other than the tolyl group is modified. Further structural modification of other parts of PF-543 may be warranted to make it suitable for medical use.

## Figures and Tables

**Table 1:** In vitro profile of PF-543 and compound **5** determined using human (HLM), dog (DLM), rat (RLM) and mouse (MLM) liver microsomal stability (% remaining during 30 min).

Compound	HLM	DLM	RLM	MLM
PF-543	6.4	7.3	7.8	9.4
**5**	7.6	5.5	6.8	9.8
